# Choosing to Provide: Early Medical Abortion and Clinician Conscience in Ireland

**DOI:** 10.1007/s10728-024-00490-2

**Published:** 2024-09-01

**Authors:** Mary Donnelly, Claire Murray

**Affiliations:** Law School, University College Cork, Cork, Ireland

**Keywords:** Abortion, Conscience, Providers’ motivation, Conscientious commitment, Conscientious objection

## Abstract

Providers are essential to the delivery of abortion care. Yet, they often occupy an ambiguous space in political discourse around abortion. The introduction of a new abortion service in Ireland invites us to look afresh at providers. Since the Health (Regulation of Termination of Pregnancy) Act 2018 came into force, by far the most common form of abortion care has been early medical abortion (EMA). This is typically provided by General Practitioners (GPs), with approximately 10% of GPs having chosen to provide EMA. This article draws on an empirical study of providers to investigate their motivations for, and experiences of, provision and their views on colleagues who have not chosen to provide. The study shows that for many providers, the choice to provide was grounded in a moral commitment to protecting women’s rights to autonomy and health and ensuring that the harms of the past were not repeated. The article argues that notwithstanding increased normalisation of EMA in Ireland, conscience still has a role to play in abortion care provision and it is important to reflect on the various aspects of this role.

## Introduction

Providers are essential to the delivery of abortion care. Yet, they often occupy an ambiguous space in political discourse around abortion. In jurisdictions with stable legal frameworks and established clinical pathways for abortion care, the contribution of providers is sometimes rendered invisible [19]. In contrast, when abortion care is politically contentious, the position of providers becomes all too obvious, and they constitute easy targets for anti-abortion activists who can inflict reputational, emotional, economic and sometimes physical harms. This has been especially evident in the United States where, as Lisa Harris shows, providers are repeatedly represented as less qualified and competent than other clinicians and as motivated by financial gain rather than patient care [24, 25]. While most of these representations come from those who oppose access to abortion, Harris also identifies portrayals of providers of ‘backstreet’ abortions as dangerous and unprincipled in pro-choice arguments in political struggles for abortion rights [25]. These negative representations of providers are often reinforced by the law, whether through the introduction of targeted restriction on abortion providers (TRAP) laws, [49] which were widely used by anti-abortion legislators in the United States prior to *Dobbs v Jackson Women’s Health Organization* [16]; a more general abortion ‘exceptionalism’ where the law treats abortion care differently to other forms of lawful healthcare [8]; or through statutory protection for conscientious objection which suggests that the only conscientious approach is to deny care [35, 41].

Developments in Ireland provide an opportunity to look afresh at the choice to provide. The Irish situation is notable for two reasons: first, meaningful abortion care provision has been lawful only since 1 January 2019 and secondly, the most common form of abortion care,^1^ early medical abortion (EMA) is typically provided by General Practitioners (GPs) rather than through specialist clinics.^2^ GPs in Ireland are independent contractors and are free to choose which kinds of lawful care they wish to offer.^3^ This means that GPs have recently had to make a choice about whether to incorporate abortion care provision into their existing practices. Approximately 10% of GPs have chosen to provide^4^ [43].

This article explores the choices around provision with a focus on the role played by clinician conscience in such choices. We understand ‘conscience’ here as meaning ‘a faculty for moral judgements and moral action that includes a set of deeply held moral beliefs and values’ [23]. In this respect, conscience has a moral, although not necessarily a religious, dimension [37]. In exploring the role of conscience, the article begins by setting out the theoretical context for understandings of conscience in abortion care. It then turns to the position in Ireland, looking first at how EMA became part of the Irish medical landscape and then at the legal framework that applies to EMA providers. Following this, the article presents the findings of an empirical study of EMA providers, which investigates their motivations for provision, their experiences of provision, and their views on colleagues who have not chosen to provide, before concluding with observations on more generally applicable lessons from the Irish experience.

## Understanding the Role of Conscience in Providing Abortion Care

Providers are central to the delivery of safe abortion care. Studies across a range of jurisdictions, [5, 6, 30, 31] show that clinicians’ decisions not to provide, sometimes presented as grounded in their legal right to conscientious objection, have a profound impact on access to care. In some jurisdictions, this has led to the development of specialist abortion care providers. In England and Wales, for example, two out of three abortions are provided by one of two charities, operating under an NHS contract: the British Pregnancy Advisory Service (BPAS) and Marie Stopes International [46]. The impact on access has also led to considerable debate on whether (and when) conscientious objection is ethically permissible [22, 23, 37, 47].

Ever since Carole Joffe’s ground-breaking study [29] of 45 ‘doctors of conscience’ in the United States prior to and after *Roe v Wade* [45], it has been recognised that abortion providers are often motivated by concerns of conscience. Joffe found that providers’ experiences of women who had been harmed by botched abortions had led to their decision to provide care notwithstanding the legal prohibition and to advocate for legal change, sometimes at considerable personal cost. Dickens and Cook adopt the term ‘conscientious commitment’ to describe this motivation. They describe this as ‘the reverse of conscientious objection’ and argue that it constitutes an important counter-narrative to legal and other presentations of conscience solely in terms of objections to providing care [15, p. 163]. Conscientious commitment brings providers into direct conflict with the criminal law or with regulatory authorities [23, 37]. As described by Carolyn McLeod, the relevant features of such services are that they are not ‘standard’ and that they are offered on the patient’s request/with the patient’s consent [37: p. 6].

While provision of lawful care does not constitute conscientious commitment in this sense, analysis from several jurisdictions indicates that moral reasoning plays a role in individual providers’ decision to provide abortion care [14, 15, 24–26, 37]. Obstetrician and abortion care provider Lisa Harris describes how, in her work in the United States, she has observed ‘abortion providers to be, as a group, people called to this work from their deepest core ethical beliefs, who feel a duty to take on care that most others—even other staunchly pro-choice colleagues—will not’ [﻿^25^, p. 199]. Contemporary research from the United Kingdom also identifies a link between a commitment to women’s health and autonomy and the decision to become an abortion care provider [32, 46, 48].

Ellie Lee and colleagues found that contemporary providers ‘communicated a strong sense of commitment to the moral worth of providing abortions, as part of medical care’ [32, at p. 31]. Providers also rejected a claim to authority in respect of decision-making about abortion, instead considering that their responsibility was ‘to provide healthcare in the form of abortion services, and this meant *upholding* decision-making by women’ [32, at p. 32, original emphasis]. However, it should be remembered that this is not necessarily always the case. Several analyses of abortion care provision in the United Kingdom in the early days following the introduction of the Abortion Act 1967 suggested that providers were motivated by a concern to enhance their professional status and reinforce their epistemic authority [34–36, 51]. Krajewska identifies a broadly similar dynamic among contemporary Polish abortion care providers [31]. Thus, it cannot simply be presumed that the decision to provide is inevitably based on a moral commitment.

While providers of lawful care do not have to contend with a direct risk of legal or regulatory sanction, the decision to provide may still come at a cost. Such costs may include disagreements with employers or colleagues; becoming a target for protests and disruption or for media or other ‘stings’ [48]; and feelings of being stigmatised in both professional and personal contexts [25, 33]. Providers may also experience internal conflicts between their general commitment to provide and provision in the specifics of an individual situation where, as Harris describes, ‘it “feels wrong” to do an abortion in the circumstances’ [25, at p. 200; see also in an Irish context [12]]. Harris points out that providers can often find it difficult to articulate these feelings, which she describes as ‘dangertalk’ because of the way, in a polarised political context, this can be used to advance anti-abortion narratives [25].

## Abortion Care in Ireland: Operationalising a New Service

Until 1 January 2019, the provision of abortion care was largely prohibited in Ireland. This was because, in 1983, the Eighth Amendment to the Constitution of Ireland introduced a specific protection for ‘the right to life of the unborn.’ This prevented any possibility of legislating for abortion in all but the most extreme circumstances [40]. Following a lengthy advocacy campaign by a wide range of bodies [21], including clinicians, many of whom were members of the advocacy organisation Doctors for Choice [1], in May 2018, almost two-thirds of those voting in a constitutional referendum (generally referred to as the ‘Repeal’ referendum) chose to replace the constitutional protection for the life of the unborn with a clause allowing provision to be made by law for the regulation of termination of pregnancy. This was delivered through the Health (Regulation of Termination of Pregnancy) Act 2018 (HRTPA). The HRTPA makes termination of pregnancy lawful where there is a risk to the pregnant woman’s^5^ life or health (HRTPA, ss. 9 and 10); where the foetus has a life-limiting condition (HRTPA, s. 11); and, without any requirement for a reason, where the pregnancy has not exceeded 12 weeks’ gestation (HRTPA, s. 12). Medical professionals are central to the operation of the HRTPA. In respect of each of the statutory grounds, one (and sometimes, two) medical professionals must certify that they are ‘of the reasonable opinion formed in good faith’ that the applicable ground is met.

The HRTPA includes several of the ‘exceptional’ elements typically associated with abortion legislation [8]. Of particular relevance to EMA is the requirement that, for pregnancies under 12 weeks gestation, there must be at least 3 days between the certification of the pregnancy by the medical professional and the commencement of the termination (where the woman takes mifepristone) (HRTPA, s. 12(3)).^6^ Other exceptional elements include specific reporting requirements to the Department of Health (HRTPA, s. 20); the potential criminal liability of anyone, including a medical professional, who ‘intentionally end[s] the life of a foetus’ outside of the framework set by the HRTPA (HRTPA, s. 23); and a specific statutory protection for conscientious objection (HRTPA, s. 22).^7^

### Developing a Model of Care

In a significant policy choice, it was decided by the Department of Health that EMA should generally be provided in the community by GPs [39]. The announcement of this mode of EMA provision met with a mixed reception among GPs. For some, especially those who had been active in the referendum campaign, this was an obvious and welcome step [39]. However, there was also a strong backlash from some GPs, who called an Extraordinary General Meeting of the Irish College of General Practitioners (ICGP) to object to the plan and some of whom walked out from this meeting in protest [9].

The Model of Care for EMA (MOC) was developed by the Health Service Executive (HSE) (the publicly funded national health and social care provider), in collaboration with various stakeholders, including the Institute of Obstetricians and Gynaecologists (IOG), the Irish College of General Practitioners (ICGP) and reproductive healthcare providers, and approved by the Department of Health [27]. The MOC contains additional elements in setting the parameters of EMA. These include that the statutory limit of 12 weeks is defined as 12 weeks + 0 days and that terminations may only be delivered in the community prior to 9 weeks + 6 days gestation. Where a pregnancy is between 10 and 12 weeks gestation or where there is a clinical indication that makes the woman unsuitable for home self-management of EMA, she is referred to secondary care.

The MOC for EMA involves three consultations, with a mandatory waiting period of three days between the first and second consultation. In April 2020, as part of emergency responses to Covid-19, the MOC was amended to allow for remote consultation (by phone or video) [50] and this MOC was placed on a permanent footing in 2023. The third consultation is a follow up, and usually involves a phone call. The MOC requires that consultations should involve advice and information about contraception. Access to service providers is coordinated through a 24/7 helpline and website called ‘My Options’ which signposts women to local providers, who may or may not have advertised their role as providers to the general public. The MyOptions service also provides information in relation to pregnancy counselling and clinical nursing supports. EMA is a funded service and it is free for those ‘ordinarily resident in Ireland’. However, the HSE only reimburses medical practitioners for abortion services if a public services number (PPSN) is provided. This means that free care is denied or delayed for some vulnerable groups, including asylum seekers and others who do not have PPSNs.

Because the EMA service was entirely new, most medical professionals (unless they had worked/trained outside of Ireland) had no experience of providing abortion care. This meant that the HRTPA and the MOC were developed in parallel and from scratch and also that GPs (and other clinicians) who were interested in becoming providers had to upskill extremely quickly [39]. Various measures were adopted to facilitate this, including a HSE-run query line for providers during the first months of operationalisation. In addition, a group of clinicians, many of whom had been involved in Doctors for Choice during the referendum campaign, established START Doctors (the Southern Task-Force on Abortion and Reproductive Topics) to provide peer support for colleagues [39].

Although the HRTPA greatly improved access to abortion care in Ireland, an independent review of the operation of the HRTPA, published in 2023, identified some significant gaps in the delivery of care [43]. At the time of the review, only 11 out of the 19 maternity hospitals in Ireland provided full termination services, resulting in an uneven geographical spread for hospital-based care services [43]. One reason for this was the exercise of conscientious objection by hospital consultants and other healthcare providers [43]. This position has improved and at the time of writing, 17 hospitals provide some termination services and the remaining 2 are expected to begin to provide in 2024. It is generally agreed that EMA provision has been the most successful aspect of the rollout to date [7, 18, 43] although here too there is considerable geographical variation, with some rural areas having very limited access [43]. Studies of non-providers indicate that the decision not to provide is more likely to be because of work-load pressures rather than a conscience-based objection [18, 39].

This overview provides the backdrop to the empirical study, to which we now turn. The study offers insights into the moral reasoning and personal experiences of providers of EMA in primary care, including the costs and benefits of their choice to provide, and their views on non-providers, in the early years of the roll-out of this service in Ireland.

## The Empirical Study

The empirical study, which obtained ethical approval from the Social Research Ethics Committee, University College Cork, explored the experiences of providers of EMA in primary care settings.^8^ The study involved semi-structured interviews with 15 providers of EMA services.^9^ Based on the existence of 413 GP contracts to provide Termination of Pregnancy (TOP) services (as of July 2022), this constitutes approximately 3.6% of GP providers at that time. These interviews were carried out by the authors on Microsoft Teams between April and September 2022. Semi-structured interviews were chosen in order ‘to bring out how the interviewees themselves interpret and make sense of issues and events’ [3]. The authors jointly developed the interview questions and guide and piloted these prior to commencing the interviews. We recruited through START Doctors and the Irish Family Planning Association, both of which shared information on the study with members.

The interviews were recorded and automatically transcribed using Microsoft Teams software.^10^ The transcripts were corrected and reviewed after each interview to address transcription errors and to ensure that any points or insights that arose could be followed up in subsequent interviews. Following this first phase of analysis, the transcripts were coded using NVivo software. Coding was agreed by consensus with each author coding an initial two transcripts followed by detailed discussion. The transcripts were then divided between the authors and subsequent coding involved ongoing discussion. Once the coding was completed, we worked together to identify, review, and refine themes, adopting a contextualist method of thematic analysis [2].

The empirical study has some limitations. First, the recruitment process employed means that respondents were self-selecting and there was a heavy representation of doctors involved in some way with the START Doctors network. Secondly, the study presents only providers’ perspectives, although in developing this article, we draw on other relevant published research to interrogate our findings and add further detail.

## Motivations for Provision

Most of the EMA providers in the Irish study identified motivations grounded in a commitment to women’s autonomy and health. Many providers recounted their personal experiences of women they had known, professionally and in some cases personally, who had had to travel abroad (typically to the UK) to access abortion care,^11^ as well as situations where women were unable to travel for financial reasons or because of their legal status (for example because they were asylum seekers/undocumented). Dr M’s^12^ description is representative:

I’ve been working for a long time in practice before the repeal of the Eighth Amendment, and just saw the appalling situations that women were in and the cruelty of it, the absolute unfairness of it, the discrimination against women who didn’t have the means to travel, like the only people I saw were the women who couldn’t get themselves on the boat and get sorted.

Several providers identified respect for human rights, women’s rights and equality. Thus, Dr H described, ‘I’m a very great believer in bodily autonomy and the rights of the individual and stuff like that. So I would really, that would be my biggest motivation around this, you know.’ This perspective, often combined with their encounters with women denied care in Ireland, had led some of the providers interviewed to become active in political campaigns to repeal the Eighth Amendment. For these providers, the decision to provide EMA was a natural and inevitable progression from their political involvement. Dr C explained the link between their political campaigning role and their decision to provide:

I was very struck by if I didn’t become a provider, what had it all been about? What had it all been for? But really, how could I look anybody else in the eye? How could I look anybody else in the eye and say, be brave, have courage. It’ll be OK. Just do it anyway. You owe this service to your patients. It’s the right thing to do. If I didn’t actually live that myself, I just would be a big fraud.

None of the providers indicated that they would have provided abortion care while it was unlawful or that they would provide abortion care outside of the limits of the HRTPA (although it should be noted that we did not specifically ask these questions). In this sense, we cannot describe the findings as examples of conscientious commitment as this is commonly understood. However, it is clear that many providers’ decisions to campaign for legal change and to provide care once this change was introduced is grounded in their moral beliefs and values.

Not all providers had been involved in the referendum campaign and some had made the decision to provide only after the service became lawful. Some of these providers saw their decision to provide as ‘pragmatic’ rather than ‘ideological’ (Dr N) and would not necessarily see themselves as ‘doctors of conscience’. Providers in this category saw the provision of abortion care as part of their professional identity as good doctors providing the full range of lawful services. For some providers, this related additionally to their identity as GPs. Dr O’C described this as follows:

I suppose when we learned about how community provision could be the gold standard and GPs would have the obvious role of family planning, and ... I just felt professionally and morally obliged to kind of step up and start providing, you know, if I could at all.

## Experiences of Provision

Perhaps inevitably, given the very significant shift in the legal and medical contexts that took place following the repeal of the constitutional prohibition on abortion, the experience of provision was multi-faceted. As we will see below, providers identified both negative and positive experiences as well as new ethical challenges arising from their decision to provide.

### Personal Experiences

Many providers described feeling nervous in advance of the service roll-out. In part, this was because of their lack of clinical experience in delivering abortion care. However, providers were also nervous about the possible reactions to their decision to provide by other people working in their practice (both clinical colleagues and other practice staff), by other patients of the practice, and about the possibility of the practice being targeted by protesters. Some providers identified stronger concerns, including anxiety about a risk of violence. As described by Dr B, ‘I think everybody had that fear that they were literally going to have people throwing stones at their windows, nearly… That was, I suppose, one of the things that was a big fear.’ Dr A spoke of contacting the police (‘the Guards’) in relation to protests outside the practice, explaining that ‘when they put the crosses up first I went around and told the guards and showed them the photographs and they said if they give you any trouble we’re there for you. So that was a very positive experience.’ The reference to ‘trouble’ here may be seen as capturing a concern that the protests could escalate into something more disruptive or violent. These fears are not surprising, given the very strong opposition to the introduction of abortion in Ireland in the past [4] and the increasingly intimidating tactics adopted by anti-abortion protesters internationally in recent years.

In general, violent or extremely intimidating protests did not materialise. However, most providers had encountered personal challenges in the initial roll-out of the service, although the level of these and their impact on the provider varied. A small number had run into serious difficulties with practice colleagues, leading one provider to leave that practice and set up on their own, and another to inform colleagues that they would leave the practice if not permitted to provide the service within the practice. In the latter case, a compromise was reached, and this provider now provides abortion care outside of practice time on the practice premises. Several providers had been the subject of protests, including in-person protests and protesters placing home-made crosses outside the practice premises. Some had also been subjected to attempted ‘stings’, receiving suspicious phone-calls, including requests by male callers for the provision of abortion care to minors.

A small number of providers identified the issue of provider stigma, especially in social and family situations, and several providers stated that they did not discuss this aspect of their work with certain family members. Dr B indicated:

But I would say there’s very few people who would talk openly about the fact that they provide that service in a social setting, because they just don’t know who’s there and what their view is going to be. And that’s actually a stigma like, that is actually, you know, a stigma that you’re carrying.

However, stigma is not identified as a significant issue for most community-based providers, a finding also supported by [11]. This may reflect the fact that providers are delivering abortion care as just one aspect of a broader practice. As Dr O’R described:

So we wear many hats, you know, and I don’t think there’s been anything, but again I don’t look at social, you know, social media and stuff like that, and some people think I might be the devil incarnate. I think when they meet me and I look after their granny, I think they’ll say actually [you are] not too bad, you know.

While the decision to provide came with costs, providers also identified positive experiences resulting from their choice. For all providers interviewed, the support of colleagues was very important in helping to overcome challenges. Given the nature of our recruitment, the support provided by START Doctors featured heavily in providers’ responses. Providers talked about how they had pooled their experiences in the early days and, as they become more experienced, were now able to help other colleagues who had more recently started to provide. As well as their greater experience, providers also described how the passage of time had led to the increased normalisation of the service. Most providers had found that, as described by Dr P, ‘providing abortions has slipped very seamlessly into what we do’.

Overall, a strong finding of the study was that the experience of provision had been extremely positive. Dr B explained:

I think that ... the reward of it is that ... you feel you are supporting that autonomy, that people are making the decision that is best for them. And the more times you hear it the more you realize that people really have thought about what is best in this situation, and quite often there’s no win for them—they’re not happy about it but they know that it’s the best thing for them.

### New Ethical Challenges

A small number of providers identified internal tensions between their commitment to provide and the actuality of provision in some circumstances. Dr P describes this tension as follows:

I think it’s important to acknowledge that we’ve made a decision that a woman should be able to have an abortion if she wants one and, big deal, you shouldn’t have to justify it or beg for one. But at the same time, like I remember, I had one girl, a student, and she had three abortions in 18 months. And I remember thinking, you know, that’s that is not what I voted yes for.

Dr O’R also referred to this as a challenge:

You know, I have had five who’ve had three. Four or five women for three. And when we’re talking on our group, we get quite paternalistic saying Jesus throw the Implanon into her. And when we had BPAS over, they were saying how dare you be so judgmental? She can have a termination every month if she wants. Still, you know, the medic part of me goes God —surely that’s tough on her, you know, but yeah—how do you reconcile that?

These tensions may also help explain the linkage which most providers drew between abortion care and contraception. As noted above, the MOC includes obligations to provide advice and information about contraception. Although we did not ask about contraception, most providers raised the issue and it was clear that they took this aspect of their duties very seriously. For most providers, the avoidance of pregnancy through contraception was preferable to termination of pregnancy.

The nature of the conversation with women about contraception varied. Dr F took a robust approach, stating that many of the women they encountered had not been using contraception and saying that they make it very clear to women that they cannot delay making a decision about contraception because ‘fertility comes back almost immediately’. However, some providers were conscious of possible shame and stigma which such conversations might evoke. Thus, Dr R indicated:

I don’t like asking them what contraception they were on because a lot of the time, they’re embarrassed to say, well, I’m on none, or whatever like and there is a bit of it, a guilt complex.

Several providers also recognised the impact of the cost of contraception and were highly supportive of the Irish Government’s programme to provide free prescription and emergency contraception to women aged 17–25 years, although they felt that these age-limits were too restrictive and strongly endorsed the roll-out of free contraception to all women of child-bearing age.^13^

## Responding to Non-Providers

As we saw above, EMA is typically provided as part of general practice. This brought many advantages, which are summarised by Dr C as follows:

I think there’s a fundamental difference. I think the local nature, the familiar nature, the slightly, my doctor wears a jumper, not a suit nature of it. It is the familiarity, so that even if it’s not in their own practice, it’s in a GP practice that they can understand the parameters of, and they can understand how it works. And rather than clinic based and also then there’s the sheer formality of walking into an abortion clinic. Even if it’s called a family planning clinic. And I think patients really appreciate the fact that you don’t know why they are there.

However, provision in general practice does mean that it falls to individual providers to make the choice to provide care. Providers of EMA are still very much a minority within broader primary care. Our interviewees all identified colleagues who had chosen not to provide for a variety of reasons. These included that their practices were already overstretched, that this kind of work did not interest them, that they were nervous about being identified as a provider and, that they had a conscience-based objection to provision. While there is limited data on the proportion of non-providers who have a conscientious objection, the general view in this regard is described by Dr S:

I think the majority of doctors that I know who don’t provide abortions would say it’s because they couldn’t be bothered or they’re too busy or they don’t want to have to do the training or, or they might have a bit of a yuck factor about it.

Most providers reported generally positive relationships with their non-providing colleagues, who referred women seeking terminations. Dr O’R explained:

Yeah, I’m probably known as the baby killer, but no, all nicely. Some of my very best friends and colleagues absolutely refused to do this. They will remain my best friends. You know I don’t do toenails. I send them up to my colleague. He sends me, you know… so we, you know, I think we’re all over it.

Dr S also recounted positive responses from non-providing colleagues: ‘And you know, usually you know you receive admiration from colleagues who felt like I didn’t feel, you know, I had the courage or whatever, but good for you. Well done.’

### Conscientious Objection

The nature of GP services means that most GPs who do not wish to provide for reasons of conscience do not have to exercise the statutory right of conscientious objection. Rather, they can simply choose not to provide. The providers in our study felt that no medical professional should be required to provide abortion care against their wishes. Reasons for providers’ support for an entitlement to conscientiously object to providing care included the importance of tolerance and respect for different views and also the more practical imperative that an unwilling provider would not provide the best quality care for women. However, several questioned the need to include a specific protection for conscientious objection in the HRTPA given that the matter was already clearly addressed by the Medical Council Guide to Professional Conduct and Ethics [38].

Providers did not address the tensions which arise between conscientious objection and access, possibly because this is less of an issue in community practice than in hospital-based care. However, several providers identified that a statutory protection for conscientious objection was stigmatising for providers. Thus, Dr B explained that ‘part of the whole conscientious objection, the way it’s set up is that somehow the people who are conscientiously objecting are better people than the people who are providing. And I think that really feeds into that, you know, stigmatizing people who are providing.’ For some providers, the absence of any statutory recognition of conscientious provision was seen as reinforcing this narrative. As described by Dr C, ‘I don’t have a particular issue with conscientious objection being protected. However, I think it gets overstated and it gets given undue importance and there’s no equivalent commitment to conscientious, you know, provision.’

While recognising colleagues’ entitlement not to provide for reasons of conscience, several providers identified examples where they had been told by women that medical colleagues or other parts of the service, eg. ultrasound providers, had obstructed women’s timely access to care or acted in a way which belittled or humiliated women seeking abortion care. This finding is replicated in other studies [7] and is identified as problematic by the Independent Review of the operation of the HRTPA [43]. For the providers we interviewed, this kind of behaviour was wholly objectionable. As described by Dr O’C:

You can’t tell a patient you can’t have an abortion. You can tell the patient I don’t provide termination care, but the duty is very much on you to say look, this is the direction you go if that’s what you need and yeah conscientious obstruction is different—it’s when you say no you can’t have an abortion because that’s wrong.

As providers recognised, obstruction of abortion care is both legally and ethically unacceptable. The HRTPA requires that anyone with a conscientious objection must ‘as soon as may be, make arrangements for the transfer of care of the pregnant woman concerned as may be necessary to enable the woman to avail of the termination of pregnancy concerned’ (HRTPA, s. 22(3)). The Medical Council Guide to Professional Conduct and Ethics goes further [38]. The most recent iteration (9th edn, 2024) states that clinicians who have a conscientious objection to providing a lawful form of care must inform the patient that they have a right to seek care from another doctor; give the patient enough information to enable them to transfer to another doctor; make such arrangements as may be necessary to enable the patient to obtain treatment and that clinicians must not ‘mislead or obstruct’ a patient’s access to care [38, at para. 42.1]. Clinicians are also required, when discussing the referral or transfer of care, to be ‘sensitive and respectful to minimise any distress’ that the clinician’s decision may cause the patient [38, at para. 42.3). While the legal and ethical position is clear, a weakness is the limited enforcement mechanisms. In sharp contrast to its criminalisation of anyone who provides abortion care outside of the framework of the legislation, the HRTPA does not include any enforcement mechanism for its obligation to transfer care [43]. A woman whose care is obstructed can make a complaint to the Medical Council; however, this is not always an easy path to pursue [42] and many women, especially if they do ultimately manage to access care, may prefer to move on.

## Conclusion

While there have been high profile curtailments of reproductive rights in recent years, there have also been some bright spots, including the liberalisation of abortion regimes in Argentina^14^ and Mexico^15^ and the Irish repeal referendum [21]. For a variety of reasons, delivery on the promise of the Irish referendum has been challenging and there is some distance still to travel to ensure full access to reproductive rights in Ireland [10, 20, 43]. Nonetheless, there has been progress, especially in the roll-out of EMA in primary care. There has been a slow but steady increase in the numbers of GPs providing abortion care [43] and the service has become increasingly normalised.^16^ An important driver of this success has been the primary care providers who chose to provide and also drove training and offered peer-support during the difficult early days. As we have seen in this article, for many of these providers, these efforts were grounded in a commitment to protecting women’s rights to autonomy and health and ensuring that the harms of the past were not repeated. As the service has become more normalised, newer EMA providers may see provision less as linked to moral beliefs and values and more as an element of the standard delivery of reproductive healthcare.

However, notwithstanding increased normalisation, conscience still has a role to play in abortion care provision. First, the link between a moral commitment to women’s autonomy and health and the decision to provide continues to be important because there is an ongoing need for new providers especially in areas where there is no local service. Secondly, notwithstanding that most non-providing GPs do not ground their decision in conscientious objection, the specific statutory protection of conscientious objection remains a relevant issue. This has the most direct impact in the hospital setting; however, it is also a contributing factor to provider stigma in all settings, through the implicit suggestion that providers are somehow less conscientious professionals than those who object to providing a lawful service. Finally, as both international experience and the study discussed here have shown, providers continue to face nuanced ethical dilemmas as they negotiate the delivery of lawful abortion care in practice. In navigating these new spaces, providers may have to reflect on their moral commitment to women’s autonomy and health, which motivated the initial decision to provide abortion care, and to recognise the ongoing importance of this commitment.

## Appendix 1: Interview Guide

### Background/Context

Can you describe the context in which you provide clinical services generally and specifically in regards to abortion care?

PROMPTS/SUBSET:

- eg the size/nature of your practice and the practice population?

- your links with and referral pathways between your neighbouring practitioners and local hospital services?

- the level of professional support/networking/isolation encountered?

- the ways in which the pathways between MyChoices (if used), and local HSE and GP services and IFPA and other organisations work?

- how regularly do you see patients seeking abortion care?

- to what extent do you publicise your provision of abortion care?

### Research Question: What Motivates Providers of Early Abortion Care to Provide this Service? (10-15 min)

INTERVIEW QUESTION:

1. Tell me about your involvement in signing up and provision of early abortion care services over the last two years?

PROMPTS/SUBSET OF QUESTIONS:

1.1 When did you decide to provide?

1.2 Why did you decide to provide abortion care (whether through a clinic or as part of your general practice or both)?

1.3 Did you have any concerns prior to your decision to provide abortion care?

1.4 What (if any) differences are there between abortion care provided by GPs as part of general practice and abortion care provided in a specialist clinic?

### Research Question: To What Extent Does the Legal Framework Impact on the Day-To-Day Provision of Early Abortion Care? (10–15 min)

INTERVIEW QUESTION:

2. To what extent does the law impact on the way you provide abortion care?

PROMPTS/SUBSET OF QUESTIONS:

2.1 What aspects of the legal framework are you most conscious of in your clinical practice?

2.2 Where do you look to for advice on legal issues?

2.3 In the context of the review of the legislation what, if any, aspects of the legal framework would you like to see amended? And why do you think those should be amended?

2.4 In what ways, if any, did the shift to remote consultation in the pandemic impact on the provision of abortion care in your experience?

2.5 Are there any other consequences that public health measures and the revised model of care had on the provision of abortion care services?

2.6 What do you think about the ‘exceptional’ features of the abortion care (when compared to other forms of healthcare)?

2.7 the provision of abortion care is legally regulated in ways that other health services are not e.g. criminal liability, statutory recognition of C.O., time limits—is this something that has an effect on your clinical practice? If so, in what way? Do you think this is necessary?

- Statutory recognition of conscientious objection
- Inclusion of specific criminal liability if the Act is not complied with
- Certification and time limits for EMA
- Mandatory 3 day wait

### Research Question: What is the Experience of Clinicians Providing Early Abortion Care? (10–15 min)

INTERVIEW QUESTION:

3 Can you describe your personal experience of providing early abortion care?

PROMPTS/SUBSET OF QUESTIONS:

3.1 Do you feel that people’s perceptions of you are affected by your role as an abortion care provider?

3.2 Have you found abortion care provision to be negatively stereotyped in Ireland?

3.3 Have you had any negative consequences (practical, professional, personal) arising from your provision of abortion care?

3.4 Do you think the legal framework contributes in any way to these negative perceptions/stereotypes/consequences?

3.5 What are the most satisfying and positive elements of your work in providing abortion care?

**Before we wrap up is there anything else you would like to add that we have not covered during the interview?**

## Footnotes

1 The vast majority of terminations (6542 out of 6666 in 2019; 6455 out of 6577 in 2020; and 4513 out of 4577 in 2021; 8042 out of 8156 in 2022; 9876 out of 10,033 in 2023) involve pregnancies of less than 12 weeks gestation [13, 43]: note that the 2021 data are not complete due to reporting problems during this year. Note also that data are not further broken down as between early terminations in primary and secondary care although the vast majority happen in primary care.

2 Abortion care is also provided through women’s reproductive health clinics such as those run by the Irish Family Planning Association [28] and the Dublin Woman Centre; however, there is only a small number of such clinics, largely in the Dublin area.

3 Where a form of care is State-funded, as is the case with termination of pregnancy, a providing GP enters into a contract with the Health Service Executive (the Irish public health and social care provider) in respect of the form of care in question.

4 It is not possible to provide exact numbers as the available data relate to the number of contracts between the HSE and primary care practices which may have multiple providers [43].

5 Adopting the terminology of the HRTPA, we use ‘woman’ here, which should be read to include people who do not identify as women and who can become pregnant.

6 This requirement, which has no clinical basis, was one of the most contentious aspects of the HRTPA and there have been repeated calls, including from an Independent Review of the operation of the HRTPA [43], for its removal.

7 This statutory protection is somewhat narrower than the equivalent provision under s. 4 of the Abortion Act 1967 (on which see *Greater Glasgow Health Board v Doogan and Anor* [2014] UKSC 68) (UK)) and applies only to a medical practitioner, nurse or midwife who carries out or participates in carrying out a termination of pregnancy.

8 Further detail on the study may be found at [17].

9 The gender breakdown of participants was 11 female providers and 4 male providers. Thirteen of the participants were in general practice and 2 were clinicians working in specialist reproductive health clinics. Of those working in general practice there was a range of practice profiles, from sole practitioners to multiple partner practices to practices with a specialism in women’s health. There was a geographical spread of providers between urban and rural locations.

10 The transcription was then checked back against the recordings by the Project Manager to ensure accuracy in the auto-transcription. All recordings were then deleted.

11 Between 1985 and 2019, approximately 75, 000 women accessing abortion care in the UK provided Irish addresses to UK providers [43].

12 All initials used are pseudonyms.

13 Since the interviews, the free contraception scheme has been extended to women aged up to 31 years.

14 Law 27.610 Access to Voluntary Interruption of Pregnancy and Post-Abortion Care 2020, which came into force in 2021, legalises abortion up to 14 weeks gestation [44].

15 This follows the decision of the Supreme Court of Mexico to decriminalise abortion: Amparo en revisión 267/2023. Ponente: Ministra Ana Margarita Ríos Farjat. Resuelto en sesión de 6 de septiembre de 2023.

16 In January 2019, there were 325 GP contracts which number increased to 422 in February 2023.
